# Clinical efficacy of transforaminal endoscopic discectomy in the treatment of recurrent lumbar disc herniation: a single-center retrospective analysis

**DOI:** 10.1186/s12891-023-06148-9

**Published:** 2023-01-12

**Authors:** Gang Xu, Xuexue Zhang, Mengye Zhu, Yi Yan, Yong Zhang, Jinjin Zhang, Fan Li, Mu Xu, Daying Zhang

**Affiliations:** 1 Department of Pain Medicine, The First Affiliated Hospital of Nanchang University, Nanchang, 330006 People’s Republic of China; 2 JXHC Key Laboratory of Neuropathic Pain, (The First Affiliated Hospital of Nanchang University), Nanchang, 330006 People’s Republic of China

**Keywords:** Transforaminal endoscopic discectomy, Recurrent lumbar disc herniation, 2 years follow-up

## Abstract

**Purpose:**

To investigate the clinical efficacy of transforaminal endoscopic discectomy (TED) in treating recurrent lumbar disc herniation.

**Methods:**

Clinical datal of 31 patients who were hospitalized in the Department of Pain Management, First Affiliated Hospital of Nanchang University, between 2015 and 2018 due to recurrent lumbar disc herniation were collected and analyzed retrospectively. Visual analogue scale (VAS) scores and Japanese Orthopedic Association (JOA) scores were used to assess alterations of patients’ leg pain intensity and nerve function, respectively. The Modified MacNab criteria were used to evaluate patients’ excellent and good rates.

**Results:**

Compared to clinical data before surgery, there was a significant reduction in VAS scores (*P* < 0.01) along with a significant improvement in JOA scores (*P* < 0.01) at 2 years after revision surgery. The patients’ excellent and good rates were 83.9% at the 2 years after surgery.

**Conclusion:**

The TED is safe and effective in the long term and is applicable to the treatment of recurrent lumbar disc herniation.

## Background

Lumbar disc herniation is a common clinical disease that causes low-back pain and lower-extremity radiating pain and is the most common type of spinal degenerative disease [1]. There are many methods to treat lumbar disc herniation in clinical practice. Clinicians often consider factors such as the size of the herniation, the adjacent relationship with the spinal cord/nerve root, the morphology of the spinal canal, the stability of the spine, and the course and severity of low-back and lower-extremity pains to choose the most appropriate individualized treatment plan for patients with lumbar disc herniation. At present, the commonly used clinical treatment strategies include conservative treatment methods such as dehydration using mannitol combined with nonsteroidal anti-inflammatory drugs (NSAIDs), analgesic symptomatic treatment, and traditional open surgery [2–4]. In recent years, with the rapid development of minimally invasive concepts and techniques, minimally invasive spinal treatment techniques such as collagenase chemonucleolysis (CCNL) and percutaneous laser disc decompression (PLDD), and especially percutaneous endoscopic spinal surgery, have gradually become common treatment measures for lumbar disc herniation [5–8].

Many clinical studies have confirmed the effectiveness and safety of endoscopic spinal techniques, especially the transforaminal endoscopic surgical system (TESSYS), in the treatment of lumbar disc herniation [9]. Unlike open surgery, TESSYS uses the natural anatomical space of the spine, i.e., the “Kambin triangle”, to establish a surgical channel, which enables less damage to the vertebral structure, less bleeding, and faster postoperative recovery [10]. The core view of spinal endoscopic techniques, including TESSYS, is to protect the normal anatomical structure as much as possible, including the nonprotruding nucleus pulposus tissue. TESSYS has the problem of difficult repair and closure of the surgical segmental annulus fibrosus tears. The presence of these factors is shared by TESSYS and traditional fenestration discectomy, and there is also the possibility of recurrent herniation of the nucleus pulposus after surgery, i.e., recurrent lumbar disc herniation. The incidence of reherniation after traditional open surgery and TESSYS is 5.2–10.2% and 3.6–10%, respectively [11–15].

Although some patients with reherniation can benefit from conservative treatment, most patients need to undergo a second surgery [16, 17]. Whether the reherniated intervertebral disc tissue is removed using the open technique or the TESSYS technique, secondary surgery faces the problems of scar adhesion and possible further damage to the bony structure of the spine [18, 19]. Compared with open surgery, TESSYS can completely or partially avoid original tissue scars by adjusting the channel approach. In addition, because of its unique characteristics, TESSYS can minimize the damage to the lamina, facet joints, and other structures that maintain the spinal stability. There have been few studies on the efficacy of TESSYS in the treatment of recurrent lumbar disc herniation. Therefore, this study investigated the clinical efficacy and safety of TESSYS in the treatment of recurrent lumbar disc herniation through a retrospective analysis.

## Materials and methods

### Clinical cases

This study was approved by the Ethics Committee of the First Affiliated Hospital of Nanchang University, and all patients provided informed consent. A total of 31 patients who were hospitalized in the Department of Pain Management, First Affiliated Hospital of Nanchang University, between 2015 and 2018 due to recurrent lumbar disc herniation were included in this study. The initial surgery of all patients is TED and they underwent TED again in this study. The inclusion criteria were as follows: 1) recurrence of lower-extremity leg radiating pain after the initial surgery; 2) relief of symptoms for more than 1 month after the initial surgery [20]**;** 3) confirmation by CT, MRI, or other imaging techniques that the herniated nucleus pulposus oppressed the corresponding nerve root, which was clinically consistent with the clinical symptoms and physical examination; 4) no significant alleviation in symptoms after conservative treatment; a 5) voluntary participation and cooperation with the follow-up. The exclusion criteria were as follows: 1) lumbar spondylolisthesis or lumbar instability; 2) structural spinal deformity; 3) history of spinal tuberculosis, spinal infection, or tumor; and 4) the presence of severe coagulation dysfunction or surgical contraindications such as severe cardiovascular, cerebrovascular, endocrine, infectious, or metabolic diseases; and 5) psychiatric symptoms that prevented cooperation with surgery and follow-up.

### Surgical procedure and perioperative management

All surgeries were performed by the same experienced surgeon in our department. Informed consent was obtained from the patients and their families before surgery, and the patients were instructed to train themselves in taking the surgical position. For patients with previous underlying diseases such as diabetes and hypertension, the blood glucose and blood pressure needed to be kept at a stable level. Antibiotics were given 30 min before surgery to prevent infection. On the day after surgery, mobilization was recommended. Patients were required to wear a waistband for protection, and activities were limited for 1 month. Patients were required to return for follow-up at 3 months, 6 months, 1 year, and 2 years after surgery for evaluation of indicators such as pain and efficacy.

**Fig. 1:**
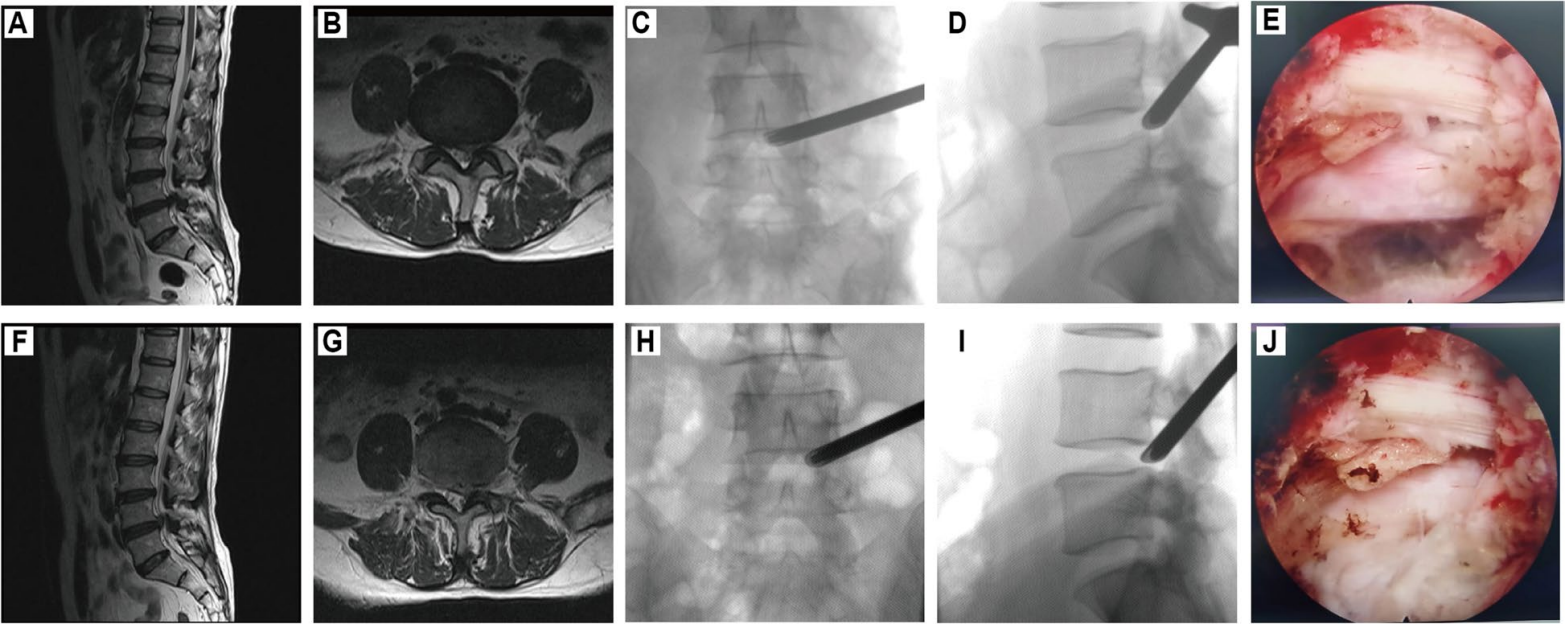
TESSYS for recurrent lumbar disc herniation. **A**, **B** Preoperative MRI of the initial surgery: L4/5 disc herniated. **C**, **D** The fluoroscopic trajectory of working cannula for removing intracanalicular herniated disc in the initial surgery. **E** The traversing nerve root is free after removal of the intracanalicular herniated disc in the initial surgery. **F**, **G** Preoperative MRI of the second surgery: L4/5 disc herniated. **H**, **I** The fluoroscopic trajectory of working cannula for removing intracanalicular herniated disc in the second surgery. **J** The traversing nerve root is free after removal of the intracanalicular herniated disc in the second surgery

During the TESSYS surgery, the patient was placed in the lateral position with the affected side facing upward, and the patient was fixed to the table at the shoulder and hip. The surgical space was determined under C-arm fluoroscopy. The puncture point was determined using the TESSYS technique (the puncture points L2/3 and L3/4 were located 8–10 cm lateral to the midline of the spinous process, and L4/5 and L5/S1 were selected 12–14 cm lateral to the midline of the spinous process). After routine disinfection and draping, a puncture needle was used to deliver 1% lidocaine (Shanghai Zhaohui Pharmaceutical Co., Ltd., China) in layer-by-layer infiltration in the puncture path. Imaging confirmed that the needle tip was located at the anterior lower edge of the superior articular process of the inferior vertebrae. A guide wire was inserted into the puncture needle, a skin incision approximately 8 mm in length was made at the midpoint of the guide wire, and the channel was dilated step by step through the dilatation tube, which was confirmed by fluoroscopy during dilatation. When the dilation tube was orthogonally located at the medial edge of the vertebral pedicle and the lateral position was located at the anterior inferior edge of the superior articular process of the inferior vertebral body, a working cannula with an outer diameter of 7.5 mm was inserted, the guide wire was removed, and an endoscope was inserted. The herniations were exposed under endoscopy, and the herniations were removed using the tools under the microscope, such as grasping forceps and a trephine. After the nerve root and dural sac were completely decompressed under the microscope, the surgical area was repeatedly washed with normal saline. When no bleeding was observed, the working catheter could be removed. The incision was sutured, and the dressing was applied (Fig. 1).

### Outcome assessment and follow-up

Preoperative and postoperative leg pains were assessed by visual analogue scale (VAS) score (0–10). Functional improvements were measured by Japanese Orthopedic Association Scores (JOA) and modified MacNab criteria. For modified MacNab criteria, “excellent” was given to patients who were free of pain, recovered normal work and performed activities without mobility limitation; “good” was given to patients whose pre-existing symptoms were almost resolved while pain occasionally existed; “fair” was given to patients who had some improvement, but still suffered from pain and/or paresthesia causing limitation of normal life; and “poor” was given to patients without any improvement and need additional treatment. Individuals were followed up at 3, 6, 12 and 24 months postoperatively.

### Statistical analyses

Data were presented as mean ± SEM and all statistical tests were performed by SPSS 17.0 statistical software (SPSS Inc., Chicago, USA). Statistical significance was assessed with unpaired t-test or one-way ANOVA. *P* < 0.05 was considered as statistically significant.

## Results

### Patients’ characteristics

Among 31 patients underwent lumbar disc discectomy in our department, 31 patients (17 male and 14 female) were diagnosed with recurrent herniation on the basis of the recurrence of sciatica symptoms and the results of MRI. These 31 patients underwent revision surgery and were included in the present study. The mean age of the recurrent patients was 56.52 ± 12.32 years (range, 35 -79 y) and the mean course of disease of the recurrent patients was 43.21 ± 55.54 months (range, 0.5—240 m) (Table 1). Among the enrolled cases, 31 underwent revision surgery at the same level (Table 2). The main recurrent disc level was L4/5 (24 patients, 77.4%), and there were 1 recurrent case in L3/4 (3.2%) and 6 cases in L5/S1 (19.4%). Most revision surgeries were conducted within 0.5 year after initial surgery.

**Table 1:** Patients’ characteristics of the recurrent group

Characteristics	Recurrent group
No. of patients	31
Age	56.52 ± 12.32
Sex	
Male	17
Female	14
Course of disease	43.21 ± 55.54
Hypertention	6
Diabetes	2

**Table 2:** LDH Characteristics of the initial and revision surgery in the recurrent group

Characteristics	Initial surgery	Revision surgery
Operation Level		
L3 – L4	1	1
L4 – L5	24	24
L5 – S1	6	6
LDH type		
Protrusion	2	2
Subligamentous extrusion	12	12
Transligamentous extrusion	17	17
Sequestration	0	0
Interval of Recurrence		
< 0.5 year		25
0.5 – 1 year		2
> 1 year		4
Modified Pfirrmann Grading		
II	8	6
III	16	16
IV	7	9

### Clinical outcomes

**Fig. 2:**
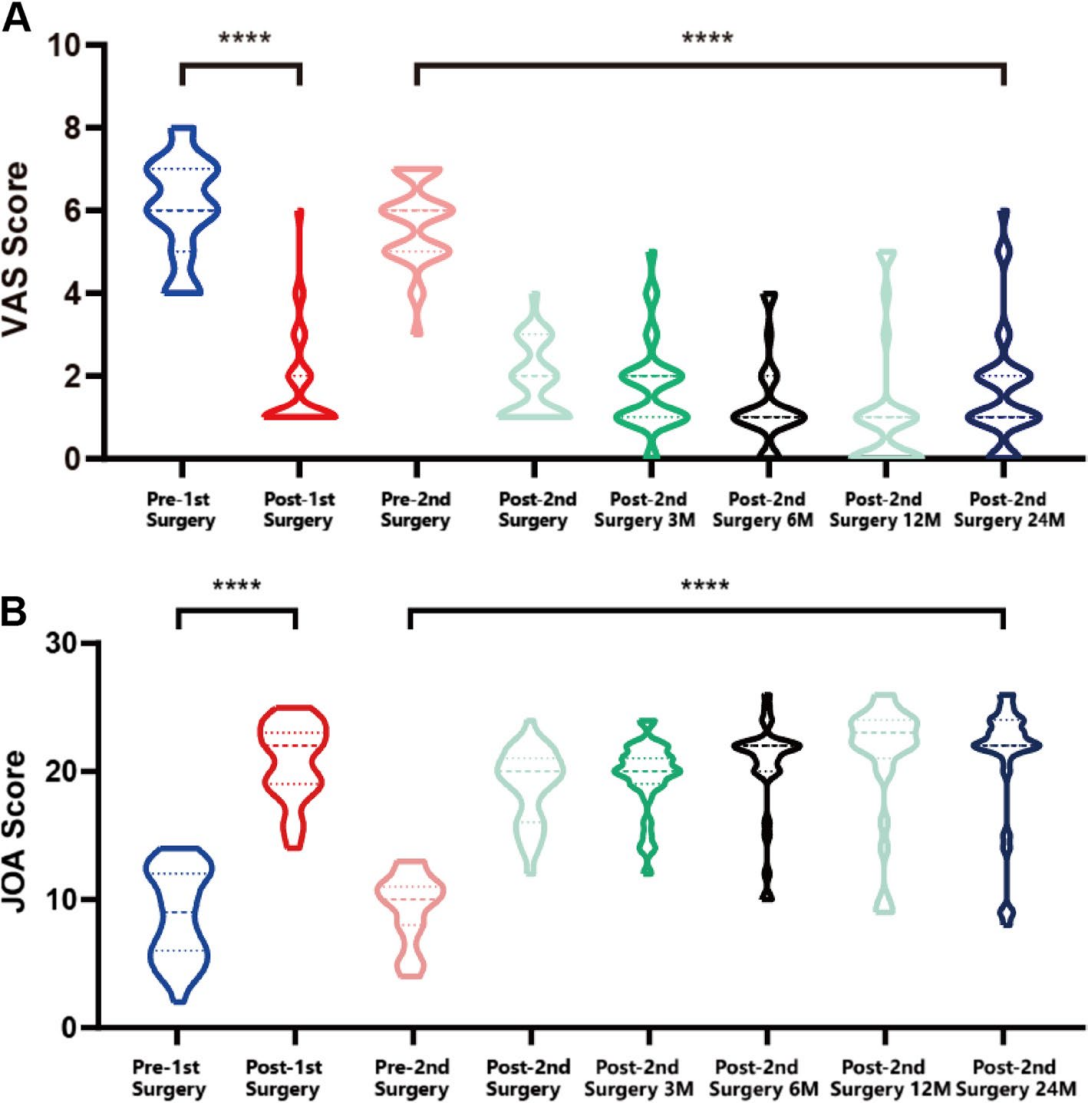
Alterations of the VAS scores and JOA scores. **A** VAS scores after revision operation were significantly decreased when compared to the value before the 2^nd^ surgery. **B** JOA scores improved significantly at the first follow-up time point after revision surgery, and the improvements remained stable to the final follow up

**Fig. 3:**
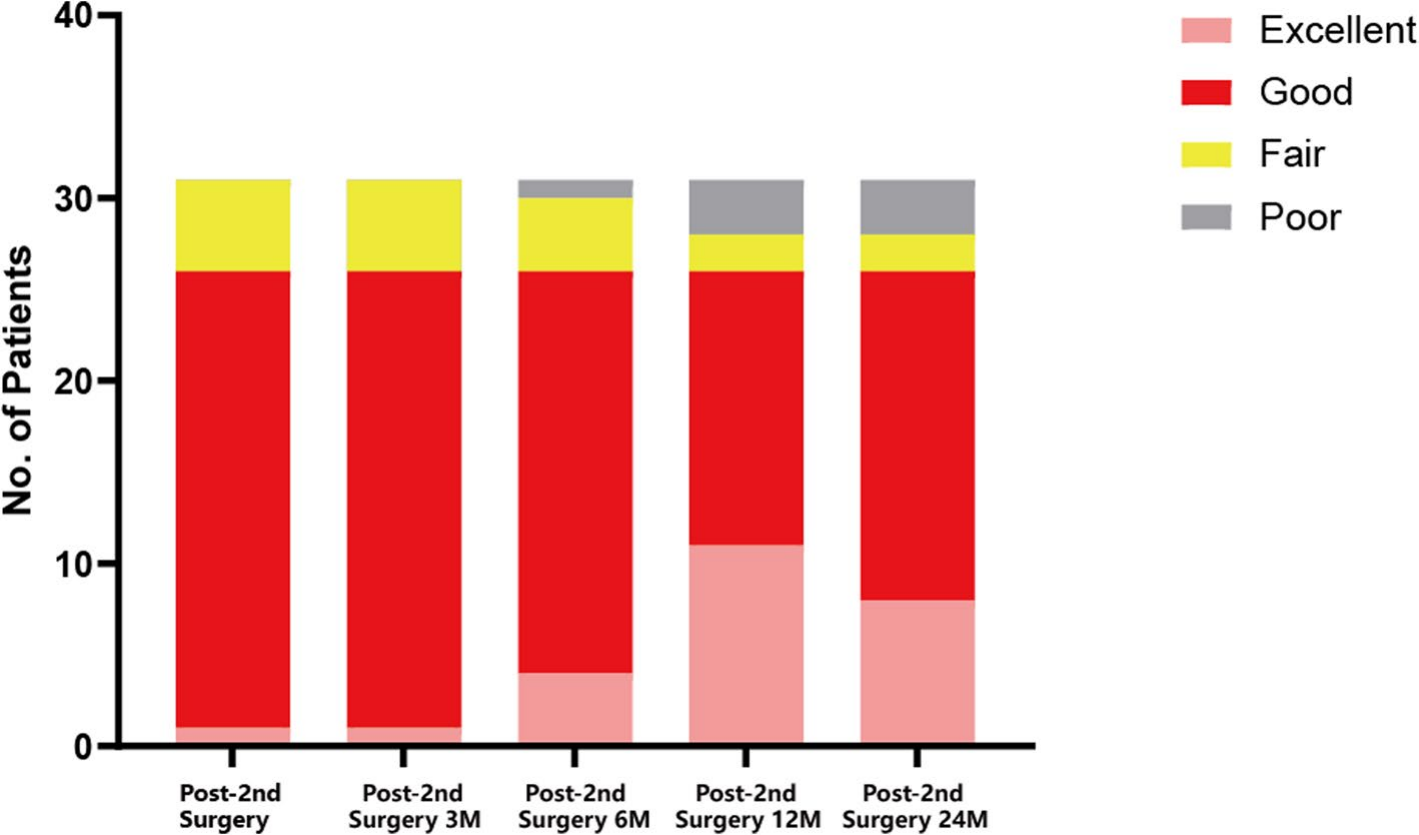
Surgical outcomes of the revision surgeries indicated by the modified MacNab criteria during the 2-year follow-up

Symptoms like leg radicular pain of the 31 patients improved immediately after revision surgery. All patients were compliant with post-operative follow-up visit, and they were followed at 3 months, 6 months, 1 year and 2 years after the revision operation. VAS, JOA scores improved significantly at the first follow-up time point after revision surgery, and the improvements remained stable to the final follow up (*P* < 0.01, Fig. 2A, B). The mean VAS, and JOA score before the revision surgery was 5.68 ± 1.01, 9.36 ± 2.70 respectively, which turn out to be 1.61 ± 1.50., 21.29 ± 4.24 at the last follow-up visit. According to the modified Macnab criteria, the surgical outcomes of the revision surgeries at the last follow-up visit were rated as follows: excellent in 8 patients, good in 18 patients, fair in 2 patients and poor in 3 patients, respectively, and the patients’ excellent and good rates were 83.9% (Fig. 3).

#### Complications

Regarding the surgical complications, no abnormal signals were observed during intraoperative monitoring in all patients. No procedure-related complications, such as nerve root injuries, epidural hematoma formation, dural laceration or delayed cerebrospinal fluid leakage occurred.

## Discussion

Recurrent lumbar disc herniation is a common cause of postoperative low-back pain in addition to postoperative scars and surgical site infections. It refers to the disappearance or alleviation of the patient’s low back and leg pain symptoms after open surgery or minimally invasive surgery, but after a time, the recurrence of nucleus pulposus herniation of the same segment of the lumbar disc is observed, which compresses the corresponding nerve root or dural sac and induces back and leg pain. For the diagnosis and treatment of recurrent lumbar disc herniation, the average cost per patient is 39,386 US dollars, which is significantly higher than that of patients undergoing conservative treatment, putting a financial burden on the patients [21]. The current definition of recurrent lumbar disc herniation is still controversial. First, some scholars hold that after the initial surgery, an asymptomatic interval of at least 6 months is needed for recurrent lumbar disc herniation to be diagnosed [22]. Alternatively, recurrent lumbar disc herniation can be diagnosed if intervertebral disc herniation recurs at the same segment after the postoperative symptoms are relieved for more than 1 month [23]. Therefore, in this study, we defined recurrent disc herniation as the recurrence of leg pain symptoms after they had disappeared or were significantly alleviated for more than 1 month after the initial surgery for lumbar disc herniation, which was confirmed by imaging of a herniated nucleus pulposus in the same segment.

The recurrent herniation of the nucleus pulposus after surgery and the recurrence or aggravation of the symptoms are correlated with body mass index (BMI), age, sex, smoking, the size of the herniation, and the degree of degeneration of the lumbar intervertebral disc [24–26]. Yao et al. [24] found that patients aged ≥ 50 years were more likely to relapse after surgery because the degree of degeneration of the lumbar intervertebral disc increases with age. After the intervertebral disc is under pressure, the nucleus pulposus may herniate again, and BMI is one of the most important risk factors for postoperative recurrence. Therefore, patients undergoing TESSYS due to lumbar disc herniation should be guided to maintain good living habits, control their body weight, and avoid excessive pressure on the spine in order to maintain the treatment effect of the surgery.

At present, recurrent intervertebral disc herniation is still treated mainly by traditional open surgery and minimally invasive interventional treatment [27–29]. Although traditional open surgery is more thorough in removing the herniation and ensuring the stability of the spine after fusion surgery, it will also lead to an increase in the surgical risk, operative time, intraoperative blood loss, and medical cost [30]. In recent years, the TESSYS technique, as an endoscopic spinal surgery method, has emerged as a new way to treat recurrent lumbar disc herniation. The TESSYS technique has a small working channel diameter, reduces the exposure of bony landmarks, does not need to damage the lamina of the surgical segment, and ensures the stability of the spine. The technique is performed under direct endoscopic vision, which facilitates the clear identification of scars, nerve roots, blood vessels, the nucleus pulposus, and other tissues. The use of local anesthesia can effectively reduce the likelihood of intraoperative dural sac and nerve root injury through timely communication with the patient. Hoogland et al. [31] reported that 262 cases of recurrent lumbar disc herniation were treated under an endoscopic transforaminal approach, and the good-to-excellent rate of this approach was 85.7%. In this study, 31 patients with recurrent lumbar disc herniation were followed up for 2 years. The good-to-excellent rate of TESSYS was 83.9%, which was similar to that reported earlier. Postoperative pain symptoms were still present, or the degree of symptom relief was low in 6.45% of patients. On the one hand, this may have been due to the presence of extensive adhesions in the original lumbar spine surgery segment during the reoperation. To avoid nerve damage and other risks, intraoperative decompression can be incomplete. On the other hand, due to the long course of the disease, long-term pain in the lower back and lower extremities can affect the structure and function of the central nervous system and promote central sensitization during peripheral nociceptive stimuli to produce chronic neuropathic pain [32]. These cause anxiety and depression, which makes the treatment effect poor.

Although the TESSYS technique, as a minimally invasive procedure, has the advantages of less trauma, less bleeding, and faster postoperative recovery, it should be noted that it has relatively limited ability to repair the annulus fibrosus with ruptured intervertebral discs, and the nucleus pulposus preserved in the intravertebral space may still become herniated again. Poor life and work habits of the patient after surgery may also lead to an increase in the degree of intervertebral disc degeneration, leading to reherniation of the nucleus pulposus. In this study, during our 2-year follow-up, no patients had recurrence of symptoms or reherniation of the nucleus pulposus. These findings may be related to their decrease in high-intensity physical activity and their avoidance of poor lifestyle habits after surgery.

This study has several limitations. Firstly, the sample size is not large enough that longer follow-up duration with more cases is needed. Secondly, we only focused on the results of TESSYS for recurrent lumbar disc herniation, the data of traditional open surgery for recurrent lumbar disc herniation should be collected and compare the TESSYS with the traditional open surgery in the treatment of recurrent lumbar disc herniation, which will be our future research focus. Finally, this study is a single-center retrospective study, a multiple center clinical study could be suggested for further study.

## Conclusions

In this study, the clinical efficacy of TESSYS in the treatment of recurrent lumbar disc herniation was investigated, the VAS score decreased and the JOA scores increased significantly preoperative to postoperative during the 2-year follow-up period. Indicating, the TESSYS technique is safe and effective in the treatment of recurrent lumbar disc herniation.

## Data Availability

The datasets used and/or analysed during the current study are available from. the corresponding author on reasonable request.

## Abbreviations

TED: Transforaminal endoscopic discectomy
TESSYS: Transforaminal endoscopic surgical system
NSAIDs: Nonsteroidal anti-inflammatory drugs
CCNL: Chemonucleolysis
PLDD: Percutaneous laser disc decompression
VAS: Visual analogue scale
JOA: Japanese Orthopedic Association Scores
